# Nutrigenomic Potentials of Phytobiotics Against Heat Stress and Allied Afflictions in Livestock Species–An In Silico Supported Review

**DOI:** 10.1002/fsn3.71788

**Published:** 2026-04-15

**Authors:** Xiaoyan Wang, Ting Yuan, Muhammad Asif Arain, Mohammed Al‐Rasheed, Aftab Shaukat

**Affiliations:** ^1^ College of Animal Science and Technology, Chongqing Three Gorges Vocational College Chongqing China; ^2^ Faculty of Veterinary and Animal Sciences, Lasbela University of Agriculture, Water and Marine Sciences Uthal Balochistan Pakistan; ^3^ Department of Clinical Sciences, College of Veterinary Medicine King Faisal University Al‐Ahsa Saudi Arabia; ^4^ Faculty of Veterinary Sciences, University of Agriculture Faisalabad Pakistan

**Keywords:** heat stress, in silico validation, livestock and poultry, nutrigenomics, phytobiotics

## Abstract

Heat stress (HS) remains a critical environmental constraint threatening the sustainability and productivity of global livestock and poultry production by including oxidative damage, compromising gut integrity, and disruption of immune homeostasis. The limited conventional nutritional strategies in conferring long‐term thermotolerance underscore the urgent need for innovative and biologically effective interventions. In this context, phytobiotics, bioactive plant‐derived compounds, have emerged as promising nutrigenomic modulators due to their potent antioxidant, anti‐inflammatory, antimicrobial, and gut‐protective properties, which collectively reinforce cellular defense mechanisms and enhance physiological adaptability under thermal challenge. This review provides a novel nutrigenomic perspective by elucidating how phytobiotics regulate the expression of key stress‐responsive molecular pathways, including heat shock proteins (HSPs), nuclear factor erythroid 2–related factor 2 (Nrf2), nuclear factor kappa B (NF‐κB), and mitogen‐activated protein kinases (MAPKs), which govern oxidative balance, immune signaling, and apoptosis. Importantly, these mechanistic insights are reinforced by emerging in silico approaches, such as molecular docking and network pharmacology, which offer predictive validation of phytobiotic bioactivity, binding affinities, and target specificity against stress‐related biomarkers, thereby accelerating the identification of high‐potential candidates. By integrating experimental and computational evidence, this review consolidates current knowledge on the genomic and physiological roles of phytobiotics in mitigating HS and associated pathophysiological disturbances, and provides an effective scientific framework for the development of sustainable, phytobiotic‐based nutritional strategies to enhance resilience and productivity in climate‐vulnerable animal production systems.

## Graphical abstract

Heat stress impairs livestock performance through oxidative stress, immune dysregulation, and reduced productivity. Phytobiotics act as natural mitigators with antioxidant, anti‐inflammatory, antimicrobial, and gut‐modulatory effects. Mechanistically, they regulate HSPs, Nrf2, NF‐κB, and MAPK pathways.

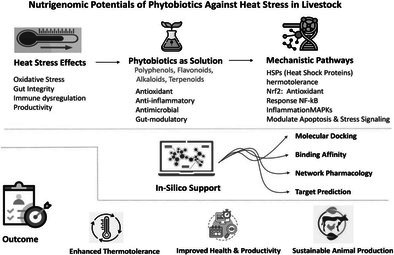

## 1 Introduction

Global livestock and poultry production is a fundamental pillar of food security and economic sustainability, providing high quality animal protein to a rapidly expanding human population while supporting the livelihoods of billions worldwide (Alagawany et al. 2020; Arain et al. 2023). However, this vital sector is increasingly threatened by climate change, particularly by the persistent rise in ambient temperatures that imposes severe HS on animals. Once considered a seasonal challenge, HS has now become a chronic, global constraint with massive economic consequences, resulting in annual losses amounting to billions of dollars due to reduced productivity, increased mortality, and impaired reproductive performance (Hassan et al. 2025; Renaudeau et al. 2012). Exposure to HS initiates a complex cascade of physiological, metabolic, and immune impairments (Inbaraj et al. 2022). Thermoregulatory responses against HS, particularly peripheral vasodilation and hyperventilation, are essential for dissipating excess heat; however, these adaptive mechanisms inadvertently compromise visceral perfusion. Hyperventilation accelerates CO_2_ elimination, leading to decreased arterial pCO_2_ and reduced blood bicarbonate (HCO_3_^−^) levels, ultimately inducing respiratory alkalosis (Lara and Rostagno 2013; Saeed, Babazadeh, et al. 2017). The resulting acid–base imbalance, combined with heat‐induced splanchnic vasoconstriction and redistribution of blood toward peripheral tissues, reduces oxygen and nutrient supply to the gastrointestinal tract (Zhang et al. 2026). This diminished splanchnic oxygenation promotes mucosal hypoxia, epithelial injury, and disruption of tight junction proteins, thereby impairing intestinal barrier integrity (Shaukat et al. 2025). Consequently, increased gut permeability (“leaky gut”) facilitates translocation of endotoxins such as lipopolysaccharide (LPS) into systemic circulation, triggering endotoxemia and exacerbating systemic inflammatory stress (Zhang et al. 2024).

At the cellular level, HS provoke significant endocrine and metabolic reprograming characterized by excessive glucocorticoid secretion, mitochondrial dysfunction, and overproduction of reactive oxygen species (ROS), culminating in oxidative stress and chronic inflammation mediated by pro‐inflammatory cytokines (Arain et al. 2025; Rehman et al. 2025). This interplay of oxidative stress, inflammation, and metabolic dysregulation suppresses feed intake, alters nutrient utilization, and impairs both innate and adaptive immune defense mechanisms. Consequently heat‐stressed animals become highly vulnerable to opportunistic infections and secondary diseases, further exacerbating productive losses (Arain et al. 2018; Nabi, Arain, Hassan, et al. 2020).

Traditionally, synthetic compounds such as antibiotics and chemical antioxidants have been used to alleviate some of the adverse effects of HS. However, increasing public concerns and strict regulatory frameworks regarding antimicrobial resistance and chemical residues have accelerated the search for safe, natural and residue‐free alternatives (Li et al. 2025a, 2025b). In this regard, phytobiotics, bioactive synergistic combinations of plant secondary metabolites such as phenolics, terpenoids and alkaloids, have emerged as promising multi‐functional feed additives with integrated physiological and immunomodulatory effects (Abd El‐Hack et al. 2025). Their benefits were initially attributed to direct antioxidant, antimicrobial, and anti‐inflammatory activities (Nabi, Arain, Rajput, et al. 2020; Saeed et al. 2021). However, advances in nutrigenomics have expanded this understanding by demonstrating that dietary phytochemicals can modulate gene expression, epigenetic regulation, and nutrient‐sensing pathways, thereby reshaping cellular responses to stressors such as HS (Arain et al. 2022; Changxing et al. 2018). This nutrigenomic framework is particularly valuable because it establishes a mechanistic bridge between phytobiotic‐driven molecular regulation, such as upregulation of antioxidant defense genes and tight‐junction proteins, and tangible physiological outcomes including enhanced thermotolerance, improved gut barrier functions and strengthened immune competence (Iliopoulou et al. 2025). Moreover, it supports the concept of precision nutrition by allowing optimization of phytobiotic type, dosage, and delivery according to species, production stage, and genetic background (Lam et al. 2025). Notably, growing experimental evidence demonstrates that targeted plant extracts attenuate heat‐induced oxidative and inflammatory markers in dairy cattle and poultry, thereby improving production efficiency under thermal stress conditions (Abd El‐Hack 2016; Saeed, Yatao, et al. 2018).

In parallel computational and in silico approaches including molecular docking, network pharmacology and transcriptomic signature matching, are increasingly being adopted to accelerate feed additive discovery and mechanistic elucidation (Sulaimany et al. 2024). These tools enable prediction of phytochemical interactions with key HS‐responsive molecular targets such as heat shock proteins (HSPs), NF‐κB, Nrf2, while also forecasting synergistic multi‐target effects within complex phytobiotic formulations (Arain et al. 2024; Safdar et al. 2024). The integration of in silico screening with in vivo validation has therefore become an effective strategy for rational phytobiotic development and for unraveling their mode of action under HS conditions.

This review focuses on both direct nutrigenomic modulation and epigenetic regulation as complementary mechanisms through which phytobiotics mitigate HS and its associated metabolic, oxidative immune, and productive impairments in livestock and poultry. Specifically, the primary aim of this review is to critically synthesize and mechanistically interpret contemporary in vivo, in vitro, and in silico evidence describing how bioactive phytochemicals regulate gene expression at the transcriptional level and modify gene activity through epigenetic processes. Particular emphases are placed on nutrigenomic regulation of stress‐responsive molecular targets including heat shock proteins, antioxidant defense regulators (Nrf2), inflammatory transcription factors (NF‐κB), and cytokine signaling pathways. Furthermore, in silico approaches, including molecular docking and network pharmacology, are integrated to strengthen target validation and predict phytochemical‐gene‐protein interactions. By integrating nutrigenomic, epigenetic, and computational evidence, this review provides a unified, mechanism‐driven framework for positioning phytobiotics as precision, next‐generation feed additives for enhancing thermotolerance, productivity, and resilience in heat‐stressed animal systems.

## 2 Heat Stress and Its Allied Afflictions in Livestock and Poultry

### 2.1 Mechanistic Basis of Heat Stress

HS, is a multifactorial physiological state triggered when environmental heat load exceeds an animal's capacity for heat dissipation, eliciting an integrated set of behavioral, neuroendocrine, metabolic and cellular responses that compromise productivity, immune competence and tissue integrity in both livestock and poultry (Brugaletta et al. 2022; Ullah et al. 2024). The mechanistic basis unfolds through a hierarchical cascade of events, initiating at the molecular and cellular levels and culminating in widespread systemic and metabolic dysfunction (Akbarian et al. 2016; Belhadj Slimen et al. 2016). At the whole‐animal level HS immediately provokes thermoregulatory behaviors (reduced feed intake, increased respiration and peripheral vasodilation) that aim to lower core temperature but also reduce nutrient intake and productive output; chronic exposure shifts energy partitioning away from growth and reproduction toward maintenance and heat loss, producing the characteristic declines in weight gain, egg production and milk yield (Brugaletta et al. 2022).

Elevated core body temperature directly compromises cellular integrity by denaturing proteins, destabilizing membranes, and altering fluidity of lipid bilayers. The most evolutionarily conserved response to this proteotoxic threat is the rapid induction of the heat shock response (HSR) (Nabi et al. 2018). This is orchestrated by the activation of heat shock factors (HSFs), primarily HSF1, which trimerize, translocate to the nucleus, and bind to heat shock elements (HSEs) in the promoter regions of target genes (Ganesan et al. 2018). This leads to the upregulated synthesis of heat shock proteins (HSPs), such as HSP70, HSP90, and HSP27, which act as molecular chaperones to prevent protein aggregation, facilitate refolding of denatured proteins, and inhibit apoptosis (Abare et al. 2023). While cytoprotective, the substantial metabolic energy expenditure required for the sustained synthesis of HSPs diverts resources away from vital productive processes like growth, lactation, and reproduction, representing a significant metabolic trade‐off (Collier et al. 2019).

### 2.2 Heat Stress and Endocrine Alteration

Elevated ambient temperature activates the hypothalamic–pituitary–adrenal (HPA) axis and sympathetic nervous system, increasing circulating glucocorticoids and catecholamines; these hormones alter appetite, glucose and lipid metabolism, and immune signaling and thereby mediate systemic effects of HS such as hyperglycaemia, lipolysis and immune suppression (Țogoe and Mincă 2024). The changes in endocrine milieu also perturb reproductive hormones, explaining reduced fertility under heat loads (Hannan et al. 2024). There is a marked reduction in the circulating concentrations of anabolic hormones, including insulin, insulin‐like growth factor‐1 (IGF‐1), thyroid hormones (T3 and T4), and leptin (Sejian et al. 2018). Concurrently, catabolic hormones like cortisol (in ruminants) and corticosterone (in poultry) are often elevated, promoting gluconeogenesis and muscle protein breakdown (Brown et al. 2021). The combined effect is a systemic shift in energy metabolism. Animals reduce voluntary feed intake (anorexia of HS) to minimize the heat increment of feeding, creating a state of negative energy balance. Consequently, the liver increases gluconeogenic activity, often utilizing amino acids from muscle catabolism as substrates, which is a primary driver of reduced growth and milk yield (Baumgard and Rhoads Jr 2013). This metabolic shift also alters lipid metabolism, leading to increased adipose tissue lipolysis and a characteristic state of non‐esterified fatty acid (NEFA) mobilization, even in the face of reduced feed intake.

### 2.3 Oxidative Stress and Alteration of Redox Balance

Hyperthermia accelerates metabolic activity and mitochondrial electron transport, thereby enhancing electron leakage and excessive generation of reactive oxygen species (ROS), a central driver of oxidative stress (Huang et al. 2026). Oxidative stress is fundamentally defined as an imbalance between ROS production and the biological system's capacity to detoxify these reactive intermediates or repair the resulting damage (Ashraf et al. 2024; Rehman et al. 2024). In modern animal production, this phenomenon represents not only a biochemical irregularity but also a major pathophysiological mechanism shaped by both endogenous metabolic processes and exogenous stressors.

Genetic selection for rapid growth and high productivity in broilers and dairy cows inherently increases mitochondrial electron leakage, promoting superoxide anion (O_2_•^−^) production as a primary ROS (Sordillo and Aitken 2009). This intrinsic vulnerability is further aggravated by external challenges, including psychological stressors (stocking density, weaning), physiological stressors (e.g., heat load, parturition), dietary pro‐oxidants (oxidized lipids, mycotoxins), and environmental pollutants (Lykkesfeldt and Svendsen 2007). During HS, antioxidant defense mechanisms are often compromised, with reduced activity or expression of key enzymatic antioxidants such as superoxide dismutase (SOD), catalase (CAT), and glutathione peroxidase (GPx) (Habashy et al. 2019). The imbalance between pro‐oxidants and antioxidants results in oxidative damage to essential macromolecules. Lipid peroxidation of polyunsaturated fatty acids disrupts cell membrane integrity, producing toxic by‐products such as malondialdehyde (MDA) and 4‐hydroxynonenal (4‐HNE) (Gallo et al. 2020). Proteins undergo carbonylation, impairing enzymatic and structural functions, while DNA damage leads to strand breaks, mutagenesis, and apoptosis (Bernabucci et al. 2010; Sordillo and Aitken 2009). Non‐enzymatic antioxidants, including vitamins E and C, glutathione (GSH), and carotenoids, are also depleted during these conditions, further exacerbating the redox imbalance (Lykkesfeldt and Svendsen 2007). Ultimately, the sustained disruption of redox homeostasis deteriorates cellular integrity and organ function, manifesting as reduced growth performance, reproductive inefficiency, immunosuppression, heightened disease susceptibility, and compromised product quality and shelf‐life (Estévez 2015). These outcomes impose substantial economic losses on animal agriculture, emphasizing oxidative stress as a pivotal constraint in livestock and poultry production systems.

### 2.4 Oxidative Stress and Gastrointestinal Barrier Dysfunction

The gastrointestinal tract (GIT) is highly susceptible to HS, as thermoregulatory adaptations disrupt its structural and functional integrity. During HS, blood is redistributed from the splanchnic bed to the skin to facilitate heat dissipation, causing intestinal hypoxia and ischemia. Subsequent reperfusion upon recovery exacerbates injury through the generation of ROS, primarily derived from mitochondrial dysfunction and enzymatic pathways such as xanthine oxidase (Akbarian et al. 2016; Kim et al. 2022). This excessive ROS production overwhelms endogenous antioxidant defenses, including SOD and GPx, while elevating oxidative stress markers such as MDA in intestinal tissues (Belhadj Slimen et al. 2016). The resultant oxidative damage impairs enterocyte integrity by oxidizing proteins and lipids, which compromises epithelial structure and function. Importantly, ROS‐mediated injury disrupts tight junction proteins such as occludin and zonula occludens‐1, leading to increased paracellular permeability—a condition widely referred to as leaky gut (Pearlin et al. 2020).

Compromised barrier function allows the translocation of luminal endotoxins, particularly lipopolysaccharide (LPS) from Gram‐negative bacteria, into the portal and systemic circulation. This endotoxemia activates toll‐like receptor 4 (TLR4)‐mediated signaling, triggering the release of pro‐inflammatory cytokines such as TNF‐α, IL‐1β, and IL‐6 (Rajput et al. 2017; Wang et al. 2019). The ensuing systemic inflammation not only amplifies oxidative stress but also contributes to metabolic dysregulation, multi‐organ dysfunction, and overall deterioration of animal health and productivity under HS conditions (Lian et al. 2020; Pearce et al. 2013).

### 2.5 Oxidative Stress and Immunosuppression

Oxidative stress is a central mediator of HS induced immunosuppression. The integrity and function of immune cells are highly susceptible to redox imbalance, as excessive ROS can damage lymphocytes and phagocytes, impair their proliferation, and disrupt cytokine production, often shifting responses toward an anti‐inflammatory, immunosuppressive profile (Lara and Rostagno 2013; Sun et al. 2017). HS downregulates key pro‐inflammatory cytokines such as interleukin‐1β (IL‐1β) and tumor necrosis factor‐α (TNF‐α), while upregulating anti‐inflammatory cytokines like interleukin‐10 (IL‐10), collectively weakening host defense mechanisms (Quinteiro‐Filho et al. 2010). This redox‐immune dysregulation operates in a synergistic manner, where the inflammation triggered by the initial immune response amplifies oxidative stress, establishing a vicious cycle that compromises animal health and productivity (Bagath et al. 2019). The resulting immunosuppressed state increases susceptibility to opportunistic infections, reduces vaccine efficacy, and aggravates economic losses in the livestock and poultry sectors (St‐Pierre et al. 2003). Dietary antioxidant supplementation has therefore been suggested as a practical approach to strengthen thermotolerance and resilience by restoring oxidative balance (Bogolyubova et al. 2022).

Beyond redox imbalance, elevated glucocorticoids and altered metabolism further aggravate immunosuppression. HS disrupts both innate and adaptive immunity, characterized by reduced lymphocyte proliferation, impaired macrophage and neutrophil phagocytic activity, and diminished cytotoxicity of natural killer (NK) cells (Rostagno 2020). Moreover, HS often drives a Th2‐skewed immune response, which weakens resistance to intracellular pathogens and contributes to subclinical disease prevalence (Umar et al. 2020). Collectively, the mechanistic basis of HS‐induced immune dysfunction lies in a complex interplay of hyperthermia‐triggered cellular stress responses, endocrine‐mediated metabolic shifts, oxidative stress, and gut‐derived inflammation. These interconnected processes culminate in immune incompetence, highlighting the need for targeted mitigation strategies to safeguard animal health and maintain productivity under thermal stress conditions.

### 2.6 Phytobiotics: Overview and Mechanisms of Action Against Heat and Oxidative Stress

**FIGURE 1:**
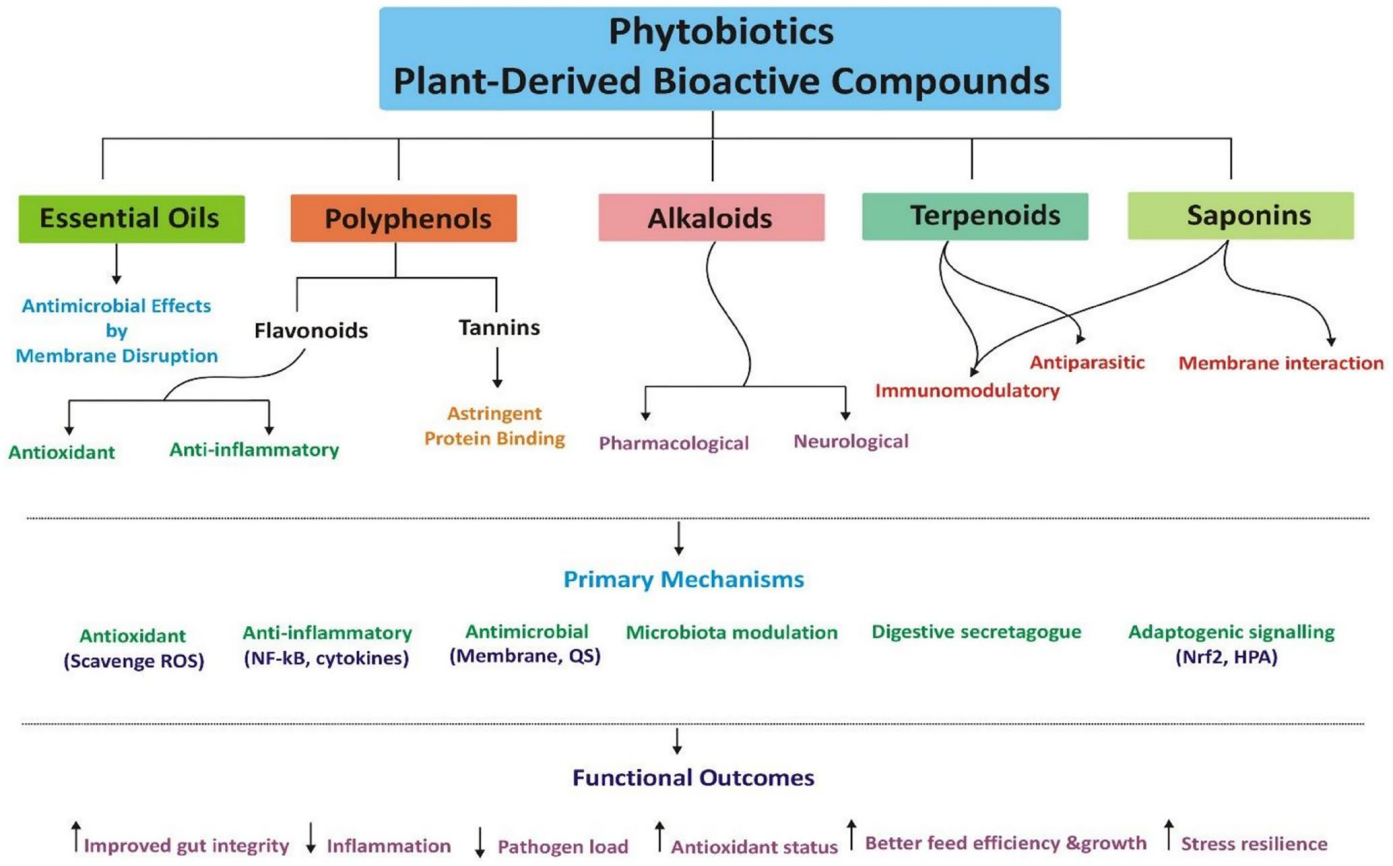
Showing the major classes of phytobiotics, their primary biological mechanisms, and the resulting functional outcomes against heat stress in animal production.

Phytobiotics, also referred to as phytogenics or botanicals, comprise a broad class of plant‐derived bioactive compounds incorporated into animal diet to enhance health, productivity, and resilience against environmental stressors (Alghirani et al. 2021; Saeed, Naveed, Arain, et al. 2017). In the context of heat and oxidative stress, their functional relevance extends far beyond growth promotion to include regulation of molecular chaperones, immune receptors, inflammatory mediators, antioxidant enzymes, lipid peroxidation indices, and intestinal barrier proteins. Their efficacy is attributed to the chemical diversity of secondary metabolites such as essential oils, polyphenols, organosulfur compounds, terpenoids, saponins, and alkaloids (Figure 1), which act through multi‐targeted and synergistic mechanisms (Vagi et al. 2005; Valenzuela‐Grijalva et al. 2017).

Under HS condition excessive generation of ROS disrupts cellular homeostasis and induces lipid peroxidation, protein denaturation, and inflammatory damage. Numerous phytobiotics have been shown to modulate antioxidant defense and suppress lipid peroxidation at the molecular level. For instance, dietary curcumin, resveratrol, oregano oil, and green tea catechins consistently upregulate the transcription and activity of endogenous antioxidant enzymes, including SOD, GPx, and CAT, through activation of the Nrf2‐ARE signaling pathway in heat‐stressed broilers, dairy cows, and pigs (Du, Zhao, et al. 2024; Liu et al. 2018; Zhang and Tsao 2016). Concurrently, these phytobiotics significantly reduce malondialdehyde (MDA) levels, a key marker of lipid peroxidation, indicating mitigation of oxidative membrane damage. For example, curcumin and thymol supplementation lowered MDA concentrations in heat‐stressed broilers and lactating cattle, reflecting improved redox balance and cellular integrity (Saeed, Naveed, et al. 2017; Zheng et al. 2023).

At the cellular stress‐response level, phytobiotics directly influence heat shock proteins (HSPs), which function as molecular chaperones safeguarding protein folding and cell survival (Hussain et al. 2021). Compounds such as resveratrol, quercetin, and withanolides from *Withania somnifera* have been shown to upregulate HSP70 and HSP90 expression in heat‐challenged poultry and ruminants, thereby stabilizing denatured proteins and enhancing thermotolerance (Iliopoulou et al. 2025; Panossian 2017). Simultaneously, these phytobiotics attenuate excessive stress‐induced apoptosis by modulating the MAPK and NF‐κB signaling cascades (Mohseni et al. 2021). Adaptogenic herbs like ashwagandha and rhodiola further modulate hypothalamic–pituitary–adrenal (HPA) axis activity, leading to lower circulating cortisol and improved cellular energy metabolism, which indirectly supports HSP‐mediated cytoprotection under thermal stress (Lopresti et al. 2022).

Phytobiotics also exert pronounced immunomodulatory effects through regulation of toll‐like receptors (TLRs) and cytokine networks. Essential oils such as carvacrol and cinnamaldehyde, along with polyphenols like curcumin and catechins, suppress overactivation of TLR2 and TLR4, thereby limiting downstream NF‐κB signaling and excessive production of pro‐inflammatory cytokines (TNF‐α, IL‐1β, IL‐6) in heat‐stressed broilers and swine (Álvarez‐Martínez et al. 2020; Du, Sarwar, et al. 2024). Concomitantly, these phytobiotics enhance the expression of anti‐inflammatory cytokines such as IL‐10, restoring immune homeostasis and preventing chronic low‐grade inflammation (Chakma et al. 2025; McGarry et al. 2024). This dual regulation of innate immune receptors and cytokines is particularly critical under HS, where immune dysregulation compromises disease resistance and performance.

Importantly, recent evidence also demonstrates that phytobiotics preserve intestinal epithelial integrity under thermal and oxidative stress by regulating tight junction proteins (Yang et al. 2021). HS induced oxidative injury downregulates Claudin‐1, Occludin, and Zonula occludens‐1 (ZO‐1), increasing gut permeability and endotoxin translocation (Xia et al. 2022). Supplementation with curcumin, thyme oil, resveratrol, and grape polyphenols has been shown to significantly upregulate tight junction protein expression and stabilize intestinal barrier function in heat‐stressed poultry, pigs, and calves by suppressing oxidative damage and inflammatory signaling in enterocytes (Lu et al. 2021; Patra et al. 2019) This gut‐protective effect synergizes with their antimicrobial activity, further reducing pathogen load and endotoxin‐driven systemic inflammation. Collectively, these findings demonstrate that phytobiotics act as molecular regulators of stress‐responsive pathways, coordinating the expression of HSPs, TLRs, pro‐ and anti‐inflammatory cytokines, antioxidant enzymes, lipid peroxidation markers, and tight‐junction proteins under heat and oxidative stress in both livestock and poultry. Through the integrated modulation of redox homeostasis, immune signaling, stress protein synthesis, and intestinal barrier integrity, phytobiotics significantly enhance thermotolerance, immune competence, and productive efficiency, reinforcing their role as sustainable nutritional tools in climate‐resilient animal production systems.

### 2.7 Nutrigenomic Concept of Phytobiotics in Animal Sciences

Nutrigenomics in animal sciences focuses on how dietary components influence gene expression and thereby modulate health, productivity, and physiological status (Müller and Kersten 2003). Unlike conventional nutrition, which primarily addresses gross nutrient requirements and deficiency disorders, nutrigenomics emphasizes bioactive compounds as dietary signals that regulate transcription, translation, and metabolite synthesis, ultimately shaping cellular and organismal phenotypes (Kussmann and van Bladeren 2011). Within this framework, phytobiotics such as flavonoids, tannins, essential oils, and carotenoids are recognized not merely as antimicrobials or growth promoters but as potent modulators of epigenetic and transcriptional processes (Lillehoj et al. 2018). Phytobiotics can influence the genome directly or indirectly by acting as ligands for key transcription factors, particularly through activation of the Nrf2 pathway, thereby inducing cellular antioxidant defense. In addition, they modulate major signaling cascades such as NF‐κB and MAPK, alter epigenetic regulation via DNA methylation and histone modification, and reshape the gut microbiota, whose metabolites further exert nutrigenomic effects (Pauletto et al. 2020; Viveros et al. 2011). This paradigm shift underpins precision feeding approaches, where phytobiotic supplementation can be aligned with genetic backgrounds to optimize nutrient utilization, enhance resilience against stress and disease, and promote sustainable improvements in animal production (Özdemir and Kolker 2016).

### 2.8 Phytobiotics as Regulators of Gene Expression Under Stress

**FIGURE 2:**
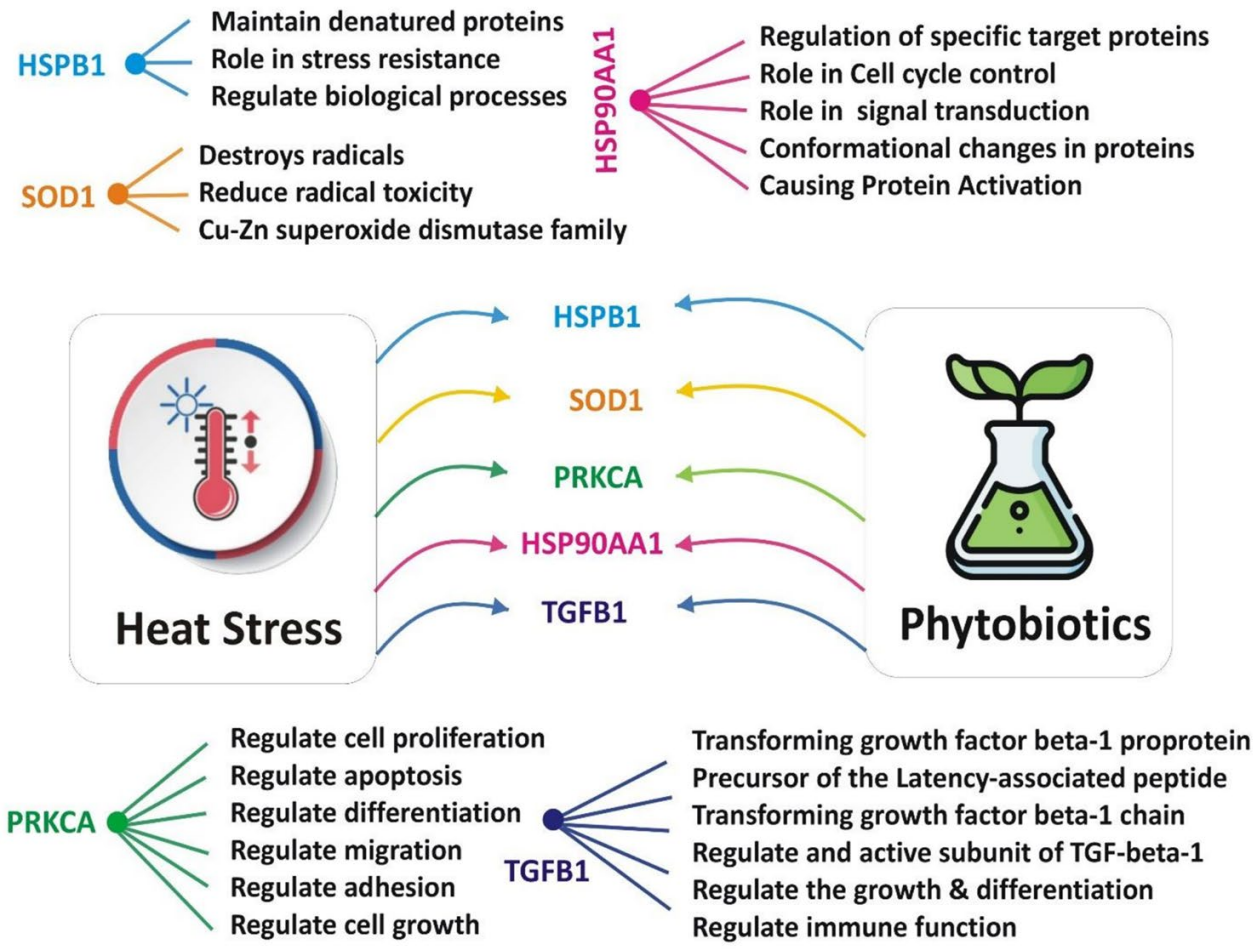
Schematic model illustrating the key molecular targets and pathways through which phytobiotics may ameliorate the impacts of heat stress.

Phytobiotics derived from herbs, spices, and plant extracts extend beyond their well‐established antioxidant and anti‐inflammatory roles to function as potent regulators of gene expression under stress. Research highlights their capacity to modulate the transcriptional landscape through three interconnected mechanisms: direct transcriptional activation, epigenetic modification, and regulation of stress‐related signaling pathways Figure 2. Evidence from animal nutrition studies demonstrates the nutrigenomic potential of phytobiotics. In broiler chickens, supplementation with phytobiotic compositions (200–400 mg/kg for 42 days) improved growth performance, meat productivity, and intestinal histomorphology, while upregulating immune‐related genes and modulating circulating myokines and microRNAs linked to oxidative stress and muscle metabolism (Chodkowska et al. 2022; Chodkowska et al. 2024; Selionova et al. 2025). Similarly, a phytogenic blend at 125–250 mg/kg enhanced resilience to cyclic HS by modulating the expression of heat shock, cytoprotective, inflammatory, and apoptotic genes in the duodenum and liver (Iliopoulou et al. 2025; Mohsin et al. 2025). In addition, phytobiotic supplementation at 100 mg/kg diet attenuated inflammasome activation and cytokine expression in broilers exposed to HS, highlighting their role in inflammatory regulation (Greene, Emami, and Dridi 2021).

Beyond poultry, phytobiotics also improve resilience in other livestock and aquaculture species. In fattening Afshari‐Shal lambs, a phytogenic‐rich herbal mixture (20 g/kg DM for 45 days) alleviated the adverse effects of HS by upregulating insulin‐related genes (IGF1, INSR) and downregulating GSK3β, reflecting improved insulin sensitivity (Hashemzadeh et al. 2023). New Zealand rabbits receiving 1 g/kg phytobiotics for 8 weeks under thermal stress exhibited improved hematological indices, immune responses, and antioxidant status, alongside upregulated immune‐related genes (Abdelmotelb and Abdel‐Monem 2025). In aquaculture, Nile tilapia fries fed a natural phytobiotic mixture (1–3 g/kg diet for 12 weeks) demonstrated improved feed utilization, whole‐body composition, and enhanced antioxidant‐related gene expression in a dose‐dependent manner (Mabrouk et al. 2025). Phytobiotics also exert epigenetic regulation of gene expression. Epigallocatechin‐3‐gallate (EGCG) from green tea acts as an inhibitor of DNA methyltransferases (DNMTs) and histone deacetylases (HDACs), resulting in hypomethylation of tumor suppressor gene promoters and transcriptional reactivation of silenced genes involved in apoptosis and cell cycle regulation (Pop et al. 2019). Likewise, resveratrol activates SIRT1, a histone deacetylase critical for stress response and longevity pathways (Singh et al. 2019). Collectively, these findings position phytobiotics as multi‐targeted modulators of gene expression that integrate transcriptional, epigenetic, and signaling‐level regulation. By enhancing stress resilience across poultry, aquaculture, and livestock systems, phytobiotics represent a powerful nutrigenomic tool for sustainable animal production under challenging environmental conditions.

### 2.9 Phytobiotics Regulate Stress‐Related Molecular Pathways

**FIGURE 3:**
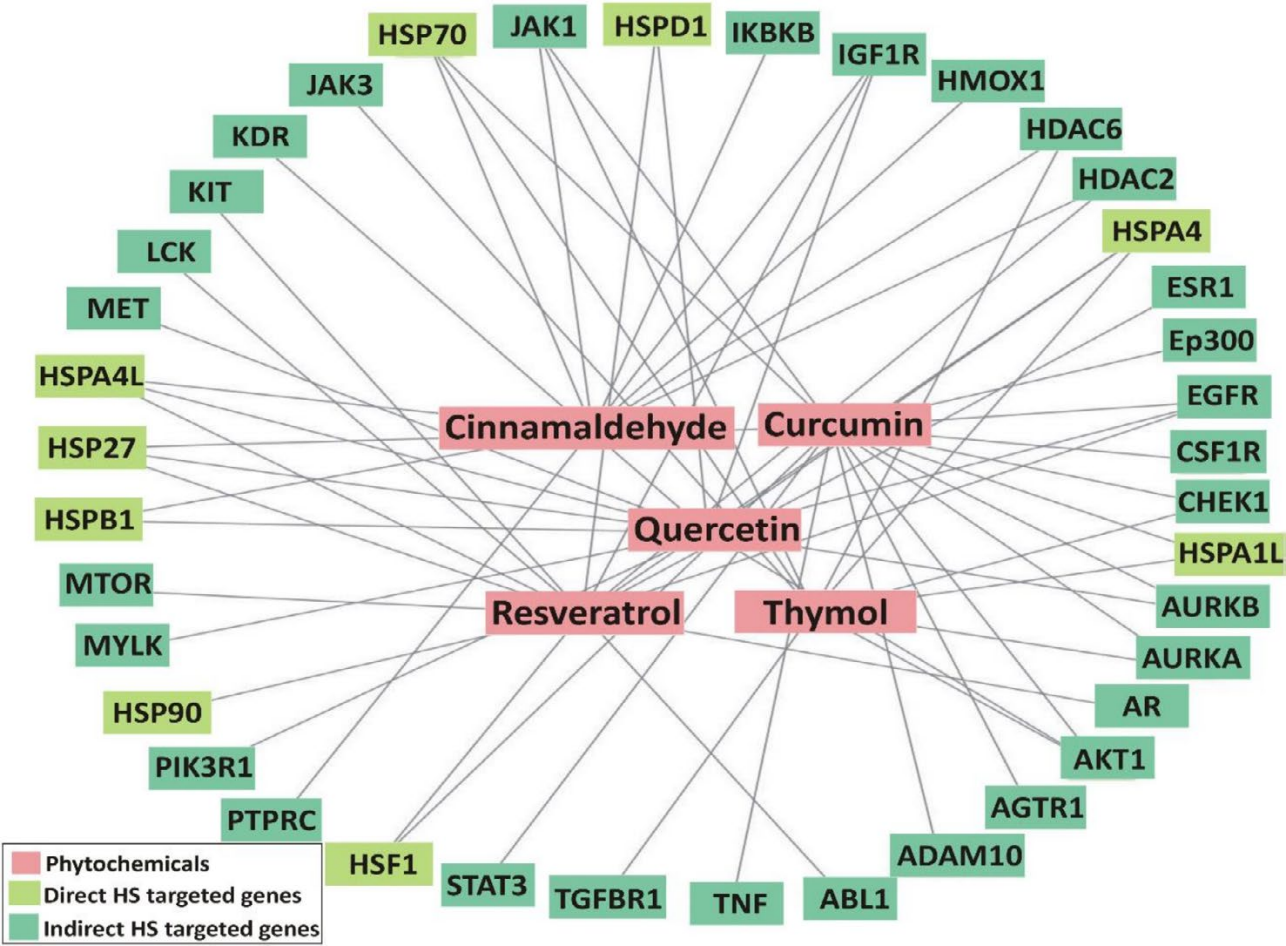
Illustrated the key molecular players in stress‐responsive pathways that are potentially modulated by bioactive compounds in phytobiotics, involved in the restoration of cellular homeostasis.

Phytobiotics have emerged as potent modulators of molecular signaling pathways governing oxidative stress, inflammation, and metabolic dysfunction in livestock and poultry (Saeed, Babazadeh, Arif, et al. 2017). The pathophysiology of stress, particularly HS, involves the dysregulation of interconnected signaling networks that disrupt redox balance, immune homeostasis, and cellular metabolism. Phytobiotics exert their protective effects through multi‐targeted nutrigenomic mechanisms that simultaneously regulate transcriptional, enzymatic, and mitochondrial functions. Recent in vivo and in vitro studies demonstrate that phytobiotics significantly influence key stress repressive mechanisms involving HSPs, NF‐κB, AMPK, PPARs, and Nrf2 (Khan et al. 2024; Li et al. 2024), as summarized in Figure 3. These concentrated molecular actions enable phytobiotics to enhance cellular resilience and restore physiological equilibrium under stress conditions. Experimental evidence strongly supports the ability of specific phytobiotics to enhance antioxidant defense and stress tolerance in poultry. Polyphenol supplementation (50–200 mg/kg for 28 days) upregulates HSP expression, protects against weaning‐induced oxidative stress, and improves gut microbial balance and intestinal integrity (Hussain et al. 2021). Similarly, dietary ginseng extract (250 mg/kg for 35 days) significantly improved antioxidant capacity, barrier functions, and HSP expression in heat‐stressed broilers (Sandner et al. 2020). Moreover, cumin essential oil 200 mg/kg for 42 days improved growth performance and feed efficiency by upregulating antioxidant and detoxification genes while suppressing pro‐apoptotic markers under HS (Yilmaz and Gul 2023). Phytobiotic blends further exhibit synergistic effects; supplementation with *Terminalia bellirica* and *Andrographis paniculata* (0.5% each for 42 days) alleviated HS‐induced behavioral disturbances, strengthened immune responses, and modulated stress‐related gene expression in broilers (Fayed et al. 2024). Similarly, a standardized phytobiotic blend (0.5 g/kg for 35 days) suppressed inflammasome activation and pro‐inflammatory cytokines during cyclic HS (Greene, Emami, and Dridi 2021). Comparable benefits have also been reported in aquatic species, where dietary dandelion extract (1–5 g/kg for 8 weeks) improved antioxidant status and intestinal morphology in fish (Tan et al. 2018).

A central thermoprotective mechanism of phytobiotics involves the modulation of heat shock protein‐70 (HSP70), a highly sensitive molecular marker of thermal and oxidative stress (Nabi et al. 2018). In chronically heat‐stressed broilers, grape seed extract (200 mg/kg) combined with vitamin C (200 mg/kg) significantly improved hematological indices and suppressed stress‐induced HSP70 overexpression, indicating reduced cellular protein damage and enhanced thermotolerance (Hajati et al. 2015). Similarly, resveratrol (400 mg/kg) restored jejunal villus architecture, increased goblet cell density, downregulated HSP70, HSP90, and NF‐κB, and upregulated epidermal growth factor in black‐boned chickens, thereby coordinating antioxidant and anti‐inflammatory responses (Liu et al. 2014). In mammals, resveratrol (20 mg/kg body weight) improved liver histoarchitecture, reduced ALT and AST levels, enhanced antioxidant enzyme expression, and suppressed NF‐κB‐mediated inflammation via HSP70 upregulation in aged heat‐stressed rats (Khafaga et al. 2019). These findings highlight the conserved cytoprotective role of phytobiotics across species.

At the mechanistic level, phytobiotics activate revolutionarily conserved redox‐sensitive signaling cascades. Bioactives such as curcumin and sulforaphane activate Nrf2 by disrupting its association with Keap1, facilitating nuclear translocation and binding to antioxidant stress elements (ARE), which induce the expression of cytoprotective genes including NQO1, HO‐1, and GSTs (Thiruvengadam et al. 2021; Tonelli et al. 2018). Thymol and carvacrol inhibit NF‐κB activation, leading to the suppression of pro‐inflammatory cytokines such as TNF‐α, IL‐1β, and IL‐6, while flavonoids such as soyasaponin downregulate ROS‐mediated PI3K/Akt/NF‐κB signaling (Liu et al. 2017; Qi et al. 2012). In parallel, phytobiotics also regulate metabolic homeostasis. Quercetin (0.05% for 42 days) reduced abdominal fat deposition and serum triglycerides in broilers through AMPK/PPAR activation (Wang et al. 2021), while salidroside (50 mg/kg/day for 12 weeks) alleviated insulin resistance and hepatic steatosis via AMPK activation and miR‐21 suppression (Almohawes et al. 2024). These findings confirm that phytobiotics exert integrated control over oxidative, inflammatory, and metabolic pathways.

Beyond systemic molecular regulation, phytobiotics exert significant effects on intestinal integrity and host microbiota interactions, which are critical determinants of stress resilience. Ginger, oregano, and clove extracts (1 g/kg for 35 days) significantly reduced *Clostridium perfringens* colonization and improved feed efficiency in broilers (Valdez et al. 2023). Gegen Qinlian decoction (18.35 g/kg/day for 14 days) alleviated ulcerative colitis in mice by restoring intestinal barrier function via AhR/IL‐22 pathway activation and modulating tryptophan metabolism (Wang et al. 2023). Phytogenic feed additives (150 mg/kg for 35 days) further enhanced ileal integrity and bacterial metabolite profiles, supporting immune homeostasis in broilers (Duangnumsawang et al. 2023). Collectively, these findings demonstrate that phytobiotics regulate stress‐related molecular pathways at multiple levels, including cellular protection, immune modulation, energy metabolism, and intestinal integrity. Their nutrigenomic efficacy is mediated predominantly through Nrf2/ARE, NF‐κB, AMPK/PPAR, PI3K/Akt, and MAPK signaling, highlighting their strong potential as sustainable natural alternatives for mitigating oxidative stress, inflammation, and metabolic disorders across animal production systems.

## 3 In Silico Approaches Supporting Phytobiotic Efficacy Against Heat Stress

### 3.1 Molecular Docking of Phytochemicals With Stress‐Related Proteins

HS triggers profound cellular disruptions, primarily by inducing protein misfolding and denaturation. Central to the defense system against such damage are heat shock proteins (HSPs), particularly Hsp90, which act as molecular chaperones to stabilize client proteins, restore their native conformation, and prevent aggregation (Schopf et al. 2017). Modulating Hsp90 activity through nutrigenomic interventions offers a strategic avenue for enhancing thermotolerance in livestock and poultry. In this context, in silico molecular docking provides valuable mechanistic insights into the potential of phytobiotics to interact with stress‐related proteins and mitigate heat‐induced cellular dysfunction.

In current screening, five phytochemicals—Curcumin, Resveratrol, Quercetin, Cinnamaldehyde, and Thymol—were selected based on their favorable bioavailability, molecular weight, drug‐likeness, and well‐documented biological activities. Their molecular formulas and PubChem CIDs are presented in Table 1 to ensure reproducibility and facilitate database referencing. Each compound was subjected to molecular docking against Hsp90, where they demonstrated variable but significant binding affinities, supporting their potential as bioactive nutrigenomic agents.

**TABLE 1:** Phytobiotics screening on the basis of active constituents.

Name of compounds	Source	Chemical structure	Molecular weight	PubChem CID
Curcumin	Turmeric	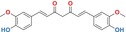	C_21_H_20_O_6_	969516
Resveratrol	Grapes, Peanuts, Berries	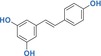	C_14_H_12_O_3_	445154
Quercetin	Onions, Apples, Citrus	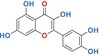	C_15_H_10_O_7_	5280343
Cinnamaldehyde	Cinnamon	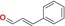	C_9_H_8_O	637511
Thymol	Thyme	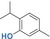	C_10_H_14_O	6989

**FIGURE 4:**
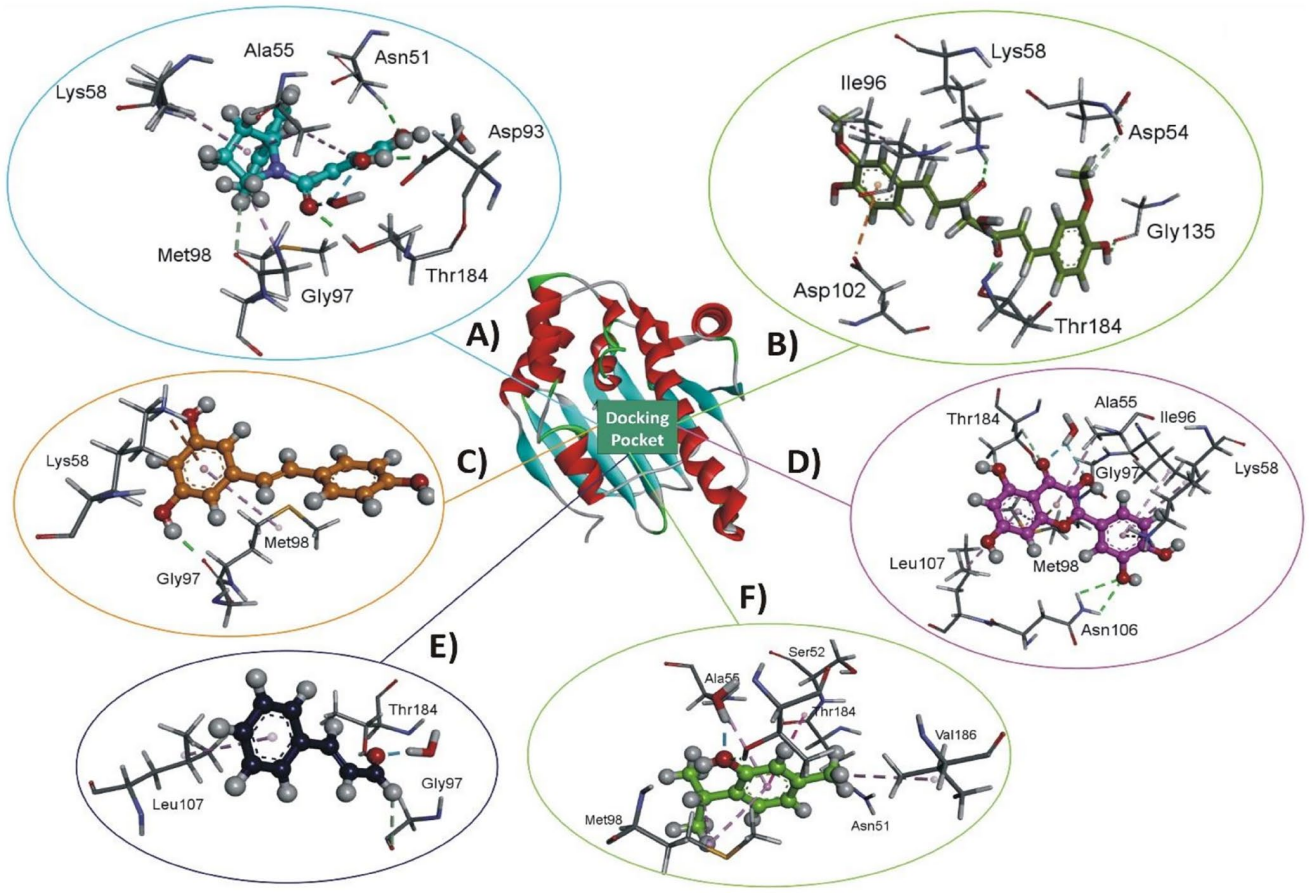
Molecular docking of phytochemicals with heat shock protein Hsp90. The central panel highlights the docking pocket of Hsp90, while individual sub‐panels represent the binding interactions of different ligands. (A) Co‐crystal ligand showing reference docking. (B) Curcumin interacting with key residues in the active site. (C) Resveratrol displaying strong affinity within the docking cavity. (D) Quercetin exhibiting the highest binding interaction with Hsp90 residues. (E) Cinnamaldehyde showing moderate binding within the docking pocket. (F) Thymol illustrating docking interactions with target amino acid residues.

Among the screened compounds (Figure 4), Quercetin exhibited the strongest binding affinity with Hsp90 (−5.966 kJ/mol). As a widely studied flavonoid, Quercetin is known for its potent antioxidant, anti‐inflammatory, and adaptogenic properties in animals under thermal stress (Abd El‐Aziz et al. 2025). Its high docking score indicates a strong potential for directly modulating Hsp90 activity. This aligns with previous in vitro evidence showing Quercetin's ability to inhibit Hsp90 ATPase activity, thereby influencing stress‐responsive signaling cascades (Bagatell and Whitesell 2004). By stabilizing protein structures during thermal insult, Quercetin may reduce apoptosis and enhance cellular survival. Resveratrol also demonstrated strong affinity toward Hsp90 (−5.466 kJ/mol). Beyond this direct interaction, Resveratrol is recognized for activating SIRT1 and promoting mitochondrial biogenesis, which strengthens antioxidant defenses and energy metabolism under oxidative stress (Marchal et al. 2013). These dual roles direct binding to Hsp90 and indirect modulation of redox pathways support its utility as a nutrigenomic intervention to enhance livestock resilience.

Moreover, Curcumin, a polyphenol derived from turmeric, exhibited moderate docking affinity (−5.434 kJ/mol). Despite its slightly lower score, Curcumin is noteworthy due to its broad biological profile. It is reported to suppress NF‐κB signaling, enhance antioxidant defenses, and modulate multiple stress‐related proteins (Agrawal and Mishra 2010; Racz et al. 2022). Its interaction with Hsp90 underscores its capacity as a multi‐target phytobiotic with synergistic potential in HS mitigation. Thymol and Cinnamaldehyde showed comparatively lower docking affinities (−4.796 and −4.081 kJ/mol, respectively). Nonetheless, their physiological relevance should not be underestimated. Thymol, a monoterpene phenol, is well established for maintaining gut integrity, enhancing immune responses, and alleviating stress‐induced dysbiosis (Hashemipour et al. 2016). Cinnamaldehyde, an aromatic aldehyde from cinnamon, has demonstrated anti‐inflammatory and antioxidative properties in heat‐challenged animals (Guo et al. 2024). Their moderate docking interactions suggest that their primary protective mechanisms may operate indirectly through modulation of gut microbiota, systemic immune responses, and oxidative balance rather than exclusively via Hsp90 binding. Collectively, these in silico findings highlight that Quercetin, Resveratrol, and Curcumin represent promising candidates with direct potential to modulate Hsp90 function, whereas Thymol and Cinnamaldehyde contribute through complementary protective mechanisms. Importantly, molecular docking serves as a predictive tool that complements but does not replace experimental validation. Factors such as compound bioavailability, metabolism, and in vivo pharmacokinetics significantly influence efficacy and must be addressed before translating these findings into feeding strategies (Bertorello et al. 2024).

### 3.2 Network Pharmacology of Phytochemicals Targeted Heat Stress Genes

**FIGURE 5:**
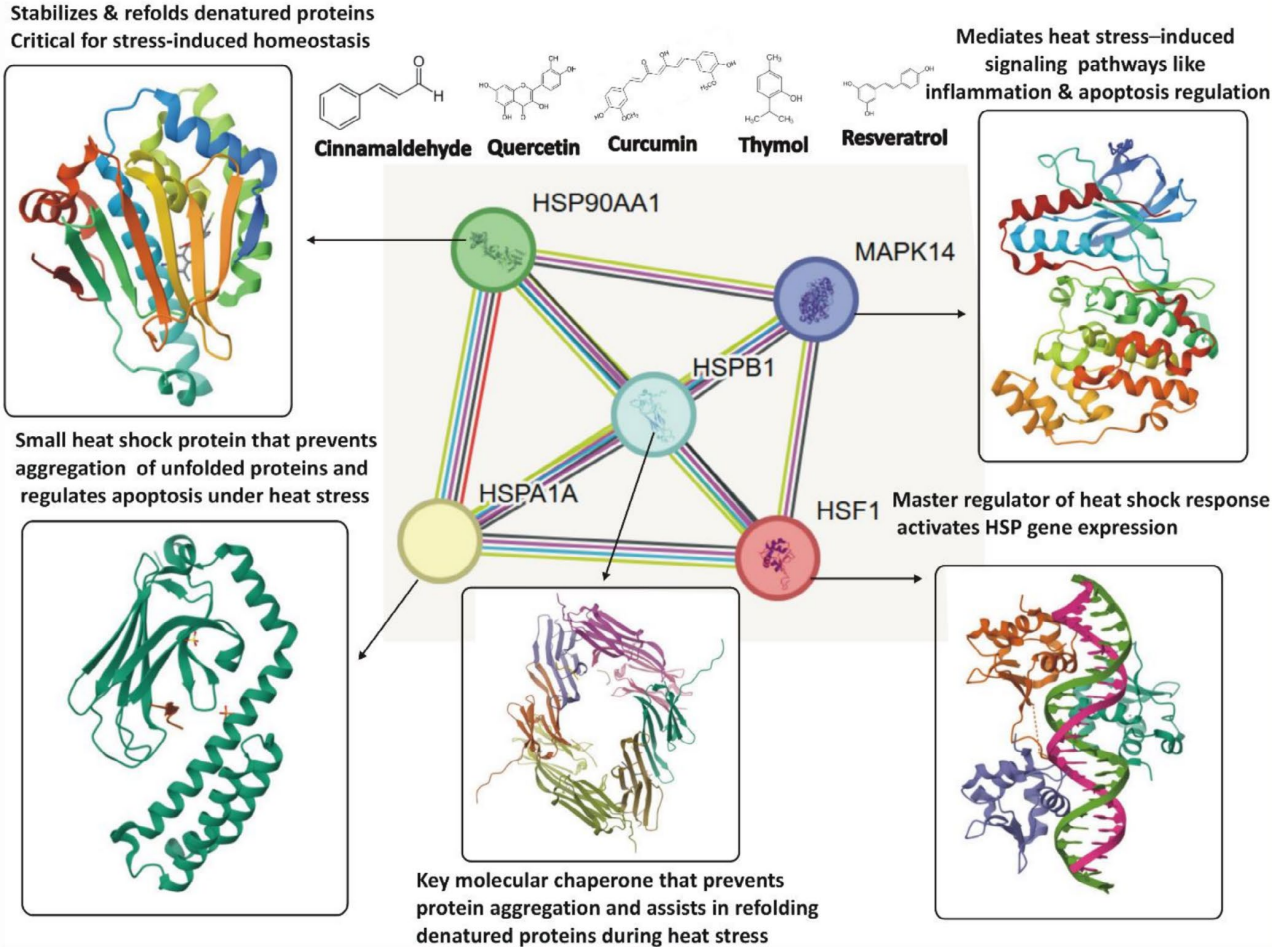
Showing the protein–protein interaction network of heat stress‐related genes targets and functional associations among key stress‐responsive proteins.

Protein–protein interaction (PPI) network analysis reveals a highly coordinated molecular defense system against proteotoxic stress, with heat shock factor 1 (HSF1), HSP70, and HSP90 functioning as central hubs Figure 5. HSF1 initiates the transcriptional activation of heat shock proteins (HSPs), while HSP70 and HSP90 act as molecular chaperones, maintaining proteostasis by refolding misfolded proteins and preventing aggregation (Richter et al. 2010). This network extends beyond these hubs, integrating smaller HSPs such as HSP27 and stress‐related kinases like MAPK14 (p38 MAPK). Crosstalk between these nodes ensures dynamic regulation: MAPK14 enhances HSF1 activity and modulates HSP27, thereby linking stress‐signaling cascades to protein refolding capacity and cytoskeletal stability (Dokladny et al. 2015).

Phytochemicals offer a promising strategy to modulate this multi‐nodal network. For example, curcuminoids from *Curcuma longa* can inhibit HSP90 ATPase activity, destabilizing HSP90‐client protein interactions while simultaneously promoting HSF1 activation and amplifying the heat shock response (Forouzanfar et al. 2019). Similarly, flavonols such as quercetin can attenuate MAPK14 signaling, reducing stress‐induced apoptosis and facilitating conditions favorable for chaperone‐mediated repair (Devi et al. 2021). Such polypharmacological actions provide a distinct advantage over single‐target inhibitors by mimicking the network's intrinsic resilience and adaptability. Collectively, the PPI topology highlights an integrated HS response module comprising HSF1‐driven transcription, HSP70/HSP90 chaperone machinery, small HSP “holdases,” and stress kinases. Targeted modulation of these hubs by phytochemicals can rebalance the system toward proteome recovery and survival rather than maladaptive cell death. Mapping compound protein interactions onto this network offers a mechanistic framework for prioritizing phytochemicals with the potential to restore proteostasis and optimize cellular resilience. This systems‐based approach underscores the therapeutic and agricultural relevance of phytochemicals in mitigating HS across biological models.

### 3.3 Phytobiotics as Modulators of Physiological and Neuro‐Endocrine Functions in Heat Stressed Livestock Species

Phytobiotics, comprising diverse bioactive plant‐derived compounds, influence multiple physiological, metabolic, and neuro‐endocrine pathways in livestock and poultry. These natural compounds act on oxidative, inflammatory, microbial, and signaling networks, enhancing systemic resilience against environmental, nutritional, and management stressors (Adetunji et al. 2025). Mechanistically, phytobiotics mitigate oxidative stress and inflammatory responses by reducing pro‐inflammatory cytokines such as TNF‐α and IL‐6 while upregulating endogenous antioxidant enzymes including SOD, CAT, and GPx (Esuola et al. 2025). Preservation of cellular integrity in key metabolic organs, such as the gut, liver, and muscle, limits stress‐induced endocrine disturbances and supports optimal energy partitioning and metabolic efficiency (Yuan et al. 2025).

A central mechanism for the systemic effects of phytobiotics is their modulation of the gut–brain axis. By strengthening intestinal barrier function, reducing peripheral inflammation, and shaping the gut microbiome, phytobiotics influence afferent signaling to the central nervous system, thereby modulating hypothalamic–pituitary–adrenal (HPA) axis activity, circulating stress hormones, thyroid function, and growth and reproductive hormone profiles (Du, Zhao, et al. 2024; Wang et al. 2022). These neuro‐endocrine adjustments enhance feed intake, nutrient utilization, and productive performance under thermal or management stress. In poultry and monogastric livestock, supplementation with phytobiotics such as oregano, thyme, turmeric/curcumin, ginger, cinnamon, and polyherbal formulations has been shown to lower plasma corticosterone or cortisol, preserve intestinal histomorphology, improve feed efficiency, and enhance both humoral and mucosal immunity (Fu et al. 2025; Saeed, Naveed, et al. 2018). At the molecular level, phytobiotics regulate NF‐κB and Nrf2 signaling and modulate neuroendocrine peptides and their receptors, fine‐tuning hypothalamic output and systemic stress responses (Cai et al. 2022).

Empirical studies further support the multifaceted benefits of phytobiotics. Turmeric supplementation in pregnant and early postpartum goats reduced oxidative stress and cortisol levels while enhancing systemic antioxidant defenses (Oderinwale et al. 2021). In broilers, curcumin alleviated corticosterone‐induced hepatic injury through improved antioxidant enzyme activity and suppressed inflammation (Shan et al. 2024). Reproductive performance is similarly enhanced; curcumin combined with PMSG increased egg production in Muscovy ducks by elevating serum estradiol and reducing the laying rest period (Gunadi et al. 2021), whereas Epimedium flavonoids counteracted glucocorticoid‐induced HPA suppression (Huang et al. 2013). Phytobiotics also mitigate age and stress‐related reproductive and metabolic decline, as shown by *Medicago sativa* in estrogen‐deficient mice (Jdidi et al. 2021). Collectively, phytobiotics integrate antioxidant, anti‐inflammatory, and hormone‐modulatory actions, supporting resilience, productivity, and overall health in livestock and poultry, positioning them as promising nutraceutical interventions for sustainable animal production.

## 4 Species‐Specific Impacts of Phytobiotics in Heat Stress Condition

### 4.1 Impact on Poultry Performance

Phytobiotics have emerged as potent natural growth promoters that significantly enhance poultry performance by modulating physiological, metabolic, and immunological functions. These bioactive compounds improve nutrient digestibility, particularly of fats and fat‐soluble vitamins, through enhanced pancreatic enzyme activity, thereby optimizing feed conversion ratio (FCR) and body weight gain. In broilers, dietary supplementation of phytobiotics at 100–300 mg/kg for 42 days consistently improved growth performance, feed efficiency, nutrient digestibility, and serum biochemical profiles compared to both antibiotic‐treated and control groups (Zaikina et al. 2022). Phytobiotics also positively influence gut health by promoting beneficial microbiota while suppressing pathogenic bacteria. For instance, supplementation at 300 mg/kg for 42 days enhanced antioxidant status, intestinal morphology, and immune responses, contributing to overall health improvement in broilers (Urban et al. 2025). Similarly, natural phytobiotic mixtures at 2 g/kg in broilers challenged with *Eimeria* spp. reduced intestinal lesions, improved microbial balance, and enhanced growth performance and feed efficiency (Galamatis et al. 2025). Inclusion at 200 mg/kg for 42 days improved villus height‐to‐crypt depth ratio, carcass yield, and gut microbial profile (Bodinga et al. 2025). On the other hand, phytobiotics also demonstrate synergistic effects with probiotics and antibiotics. Broilers supplemented with oregano essential oil (300 mg/kg), *Bacillus subtilis* (1 × 10^8^–10^9^ CFU/kg), or avilamycin (10 mg/kg) for 35 days exhibited improved growth performance, carcass traits, intestinal morphology, and meat quality, while reducing pathogenic bacterial load and protecting against necrotic enteritis (Hussein et al. 2020; Ren et al. 2019).

In laying hens, Allium‐based phytobiotics at 150–1000 mg/kg or Alquernat Nebsui L (0.5 mL/L) over 8–12 weeks enhanced egg production, egg mass, feed efficiency, and egg quality, including yolk color, shell thickness, and Haugh unit. These improvements were accompanied by favorable modulation of ileal and cecal microbiota, enhanced ovarian and oviduct development, and reduced pathogenic bacteria (Chudak et al. 2024; Marwi et al. 2021; Ruesga‐Gutiérrez et al. 2022). Phytobiotics also mitigate disease‐related challenges. Essential oil supplementation at 100 mg/kg in laying hens infected with *Salmonella* enhanced egg production, modulated cecal microbiota, improved immune responses, and upregulated stress‐ and immunity‐related gene expression, thereby supporting resilience against pathogenic stress (Laptev et al. 2021). Additionally, combinations of phytobiotics with postbiotics further improved growth performance, gut morphology, and skeletal development, demonstrating their integrated role in poultry health and productivity (Doski and Kareem 2023). In summary, these studies highlight that phytobiotics enhance poultry performance through multifaceted mechanisms involving improved nutrient utilization, gut health, immune modulation, and antioxidant defense, offering a sustainable alternative to conventional growth promoters.

### 4.2 Impact on Cattle and Buffaloes Performance

Dietary supplementation of cattle and buffaloes with phytobiotic‐rich herbal additives has demonstrated substantial benefits for growth performance, nutrient utilization, rumen function, milk production and overall health. In dairy calves, dietary supplementation of herbal extract mixture at 10 g/day for 60 days significantly enhanced body weight gain, feed efficiency, and nutrient digestibility, while positively modulating blood metabolites, rumen fermentation, and beneficial microbial populations (Jahani‐Azizabadi et al. 2022). Similarly, Holstein cows receiving a phytobiotic mixture at 50 g/day for 60 days exhibited improved milk yield, feed efficiency, and udder health, particularly in cows with high somatic cell counts, alongside reductions in plasma haptoglobin and enhanced antioxidant capacity (Hashemzadeh‐Cigari et al. 2014). Phytobiotic additives, rich in secondary metabolites such as tannins, saponins, alkaloids, and essential oils, have consistently been reported to improve ruminant productivity by reducing methane and nitrogen emissions, enhancing rumen fermentation efficiency, and improving product quality. Their bioactive properties including anthelmintic, antioxidant, antimicrobial, and immunomodulatory activities contribute to improved health and resilience under both conventional and HS conditions (Ahmed et al. 2024).

In water buffaloes, supplementation with mixed phytogenics at 20 g/day for 60 days altered rumen bacterial composition, favoring fiber‐digesting microbes, and improved the milk fatty acid profile by increasing unsaturated fatty acids while reducing saturated fatty acids (Hassan et al. 2020). Similarly, buffalo calves receiving a phytogenic composite at 5 g/kg feed for 90 days exhibited enhanced immune response, antioxidant status, nutrient utilization, and growth performance, along with reduced methane emissions (Kumar et al. 2022). In vitro studies further support these effects, where plant secondary metabolite blends (0.5%–2%) reduced methane production and increased total volatile fatty acids in buffalo rumen fluid in a dose‐dependent manner (Singh et al. 2017).

Additional interventions include synbiotic supplementation combining *Cichorium intybus* root powder (5 g/day) with Lactobacillus strains, which improved growth performance, feed efficiency, and gut health in Murrah buffalo calves by enhancing beneficial microbial populations and suppressing pathogens (Singh et al. 2021). Likewise, neem ( *Azadirachta indica* ) leaves (200 mg/kg body weight for 4 weeks) reduced gastrointestinal nematode egg counts and improved weight gain and milk yield in lactating cows (Moniruzzaman et al. 2022). Supplementation with herbs such as plantain and lemongrass enhanced dry matter intake, milk yield, milk solids, and immunoglobulin levels while reducing liver enzymes (Rahman et al. 2024). Polyherbal additives containing vitamin C (from Emblica officinalis and *Ocimum sanctum* ) improved milk production, reproductive performance, antioxidant status, and immune response in Holstein cows under HS (Mendoza Martinez et al. 2022). Finally, supplementation with *Punica granatum* and Tecomella undulata (50 g/day for 90 days) enhanced nutrient utilization, growth performance, and reduced enteric methane emissions in Murrah buffaloes without adverse effects on health (Hundal et al. 2019). Conclusively, these studies demonstrate that phytobiotic supplementation is a promising strategy to improve growth, production, metabolic status, gut health, and environmental sustainability in cattle and buffaloes.

### 4.3 Impact on Sheep and Goat Performance

Dietary supplementation with phytochemicals, prebiotics, synbiotics, and herbal feed additives has demonstrated significant benefits for sheep and goats across growth, production, metabolic, and immune parameters. In pregnant does, a phytochemical and Lactobacilli blend at 10 g/day for 60 days enhanced body weight, feed intake, nutrient digestibility, ruminal fermentation, antioxidant defenses, and the expression of immunity‐ and metabolism‐related genes (Gabr et al. 2025). Similarly, supplementation of dairy ewes with natural antioxidants at 10 g/day for 60 days improved milk yield and composition, increased oxidative stability, and positively influenced udder health by reducing somatic cell count and oxidative stress markers (Karageorgou et al. 2023). Growth performance, feed efficiency, and nutrient digestibility in lambs were significantly enhanced by prebiotic, synbiotic, and phytobiotic supplementation for 60 days, alongside improved blood metabolites and overall metabolic and health status (Saravani et al. 2025). Crossbred sheep receiving a mineral‐phytobiotic mixture at 5 g/day for 90 days exhibited increased live weight gain, carcass yield, and meat quality, reflected in higher protein content and reduced fat deposition (Perig and Kyryliv 2023). In vitro supplementation of sheep rumen with saffron petal extract (50–200 mg/L for 24 h) enhanced energy and nitrogen metabolism, increased volatile fatty acid production and ammonia assimilation, and improved antioxidant status by elevating superoxide dismutase and catalase activities while reducing lipid peroxidation (Akbari Shooshood et al. 2024).

Lambs supplemented with a polyherbal feed additive at 2 g/day for 60 days showed significant improvements in body weight gain, feed efficiency, and blood biochemical parameters, including increased total protein and reduced cholesterol levels (Razo Ortiz et al. 2020). In dairy goats, herbal feed additive at 5 g/kg of feed for 8 weeks increased intestinal lactic acid bacteria counts and promoted gut microbial balance without adverse health effects (Foksowicz‐Flaczyk et al. 2022; Jin et al. 2025). Quercetin supplementation at 0.5 g/kg for 60 days in Hu sheep during summer enhanced growth, antioxidant status, serum metabolites, and testicular development (Wan et al. 2024), while abomasal quercetin at 100 mg/kg body weight for 28 days mitigated inflammation in high‐grain‐fed goats by downregulating matrix metalloproteinase gene expression (Guo et al. 2018). Furthermore, lycopene supplementation improved metabolic health and oxidative status. Feedlot Bamei lambs receiving 100 mg/kg for 60 days exhibited enhanced plasma lipid profiles, reduced lipid peroxidation, and improved antioxidant defenses (Jiang et al. 2015). Pregnant ewes supplemented with lycopene combined with corn at 4 mg/kg body weight for 60 days showed increased circulating IgG levels in both ewes and their lambs, indicating enhanced maternal and neonatal immunity (Fallah et al. 2021). The effects of phytobiotics on physiological, reproductive, and oxidative parameters in various livestock species under HS are summarized in Table 2.

**TABLE 2:** Showing the effects of phytobiotics on physiological, reproductive, and oxidative parameters in livestock species under heat stress.

Species/breed	Phytobiotic/compound	Dose of supplement	Duration of trial	Results/key findings	Mechanism of action	References
Broiler chicken	Phytogenic feed additive	Not specified	HS model (35 days)	Improved performance traits, enhanced serum neopterin levels, and increased cutaneous basophil hypersensitivity response under HS	Immunomodulatory effects, antioxidant activity, stress alleviation	(Marimuthu et al. 2020)
Broiler chicken	Herbal adaptogen feed‐additive	As per manufacturer's recommendation	Not specified	Improved growth performance, enhanced carcass parameters, altered muscle amino acid profile under HS conditions	Modulation of stress response, antioxidant activity, improved nutrient metabolism	(Greene, Maynard, et al. 2021)
Laying hens	Ginger powder + Chinese herbal medicin	Ginger: 0.5% of feed; Herbal mix: 0.5% of feed	4 weeks	Improved production performance, enhanced serum metabolite profile, increased antioxidant status under HS	Antioxidant activity, improved nutrient metabolism, stress mitigation	(Ibtisham et al. 2019)
Layer chickens	Herbal liquid anti‐stressor product	As per manufacturer's recommendation	Not specified	Improved production performance, reduced HS indicators	Likely antioxidant and adaptogenic effects, modulating stress response	(Jadhav et al. 2014)
Cow/Early lactating	Herbal supplements	Not specified	During early lactation under HS	Improved milk yield and quality; modulation of blood parameters including antioxidant status and metabolic profile	Antioxidant, anti‐inflammatory, and metabolic regulation under HS	(Saleh et al. 2023)
Cow/Late‐lactation	Fermented Chinese herbal medicines	Not specified	Not specified	Improved milk yield and composition; enhanced immune function under HS	Modulation of immune response; antioxidant effects; alleviation of HS–induced physiological stress	(Shan et al. 2018)
Dairy calves	Mixture of phytobiotic‐rich herbal extracts	Not specified	60 days	Improved growth performance, enhanced blood metabolites (e.g., total protein), altered rumen fermentation (increased VFA), and modulated bacterial population	Antioxidant, antimicrobial, and rumen microbial modulation; improved nutrient utilization	(Jahani‐Azizabadi et al. 2022)
Water Buffaloes	Mulberry leaf flavonoids	Not specified	60 days	Improved milk production, enhanced antioxidant status, improved metabolic profiles	Antioxidant, metabolic regulation, modulation of oxidative stress	(Li et al. 2020)
Sheep/Hu	Quercetin	Not specified	Summer feeding period	Improved growth performance, enhanced blood parameters, promoted testicular development	Antioxidant, anti‐inflammatory, supports reproductive physiology	(Wan et al. 2024)
Lambs	Naringin	Not specified	Not specified	Improved productive performance, enhanced antioxidant status, and modulated immune response under HS	Antioxidant and immunomodulatory effects	(Alhidary and Abdelrahman 2016)
Goat	Flavonoid‐enriched supplementation	Not specified i	Prenatal period	Improved growth performance, reduced effects of prenatal stress in kids reared under sub‐tropical conditions	Antioxidant, anti‐stress, and potential modulation of stress‐related metabolic pathways	(Yaseen et al. 2022)
Damascus Goat bucks	Phytochemicals	Not specified	Not specified	Improved haemato‐biochemical parameters, enhanced oxidative status, better semen quality, and positive histological changes under HS	Antioxidant activity, modulation of blood biochemistry, protection of tissue integrity	(Abd El‐Hamid et al. 2023)

## 5 Challenges and Limitations

Despite their promising nutrigenomic potential, the practical application of phytobiotics in livestock and poultry production faces multiple scientific and technical challenges that hinder widespread adoption. One of the primary limitations is the inherent variability in phytobiotic composition and bioavailability. The phytochemical profile of plant‐derived products is influenced by plant genotype, geographical origin, climatic conditions, harvest timing, and post‐harvest processing (Bento et al. 2013). This variability results in batch‐to‐batch inconsistency, leading to unpredictable biological responses and complicating the replication of positive outcomes. Additionally, the bioavailability of active compounds affected by solubility, gastrointestinal stability, and interactions with the gut microbiota varies significantly among species and individuals, further complicating dose optimization (Zeng et al. 2015). The absence of standardized dosages and formulations represents a second major challenge. Without consistent chemical profiling, determining effective and safe doses for different species, production stages, or stress conditions remains largely empirical. Current recommendations are often extrapolated from small‐scale studies and lack the robust validation provided by large‐scale, controlled trials. Moreover, developing stable, palatable, and shelf‐stable phytobiotic feed formulations that preserve active ingredients poses a substantial technical challenge for the feed industry (Dhama et al. 2015).

A critical scientific gap exists in the limited use of omics‐based and in silico validation in target farm animals. While rodent studies and computational models offer valuable preliminary insights, their predictive reliability is limited for polygastric livestock and commercial poultry due to species‐specific differences in digestive physiology, metabolism, and microbiome composition (Mishra and Jha 2019). Targeted transcriptomic, proteomic, and metabolomic studies under real‐world stress conditions are essential to move beyond correlation and establish causal mechanisms, thereby validating computational predictions (Kim et al. 2025). Finally, regulatory and safety considerations constrain commercialization. In many regions, phytobiotics occupy a regulatory gray zone between feed additives, nutraceuticals, and drugs. Clear guidelines for efficacy, safety including residues in animal products and quality control are often lacking. Comprehensive evaluation of potential drug interactions, long‐term administration effects, and impacts on antimicrobial resistance is critical to ensure consumer safety and regulatory compliance (Additives et al. 2019). Addressing these multifaceted challenges through coordinated research and industry collaboration is crucial for translating the theoretical nutrigenomic benefits of phytobiotics into practical, reliable, and sustainable strategies for mitigating HS in animal agriculture.

## 6 Future Prospects and Research Directions

The emerging field of phytobiotic nutrigenomics offers transformative potential for mitigating HS in livestock. Future research should adopt a systems biology framework, moving from generalized supplementation to precision nutrition strategies. Central to this approach is precision nutrigenomics, which identifies genetic markers, such as SNPs in heat shock protein genes (HSP70, HSP90), that influence susceptibility to HS and responsiveness to specific phytobiotics. This enables the development of effective feed additives optimized for an animal's genotype, breed, and production system. The exploration of phytobiotic chemical diversity can be accelerated through *in‐silico* tools powered by Artificial Intelligence and Machine Learning, predicting bioactive compounds, bioavailability, molecular interactions with host targets (e.g., NF‐κB, Nrf2 pathways), and gut microbiome modulation. Integration with multi‐omics approaches including genomics, transcriptomics, and metabolomics will elucidate molecular responses to HS and phytobiotics, uncover robust biomarkers, and identify synergistic phytocompound combinations. Converging precision nutrition, AI‐driven discovery, and multi‐omics validation promises next‐generation, evidence‐based phytobiotic solutions that enhance thermotolerance, productivity, animal welfare, and environmental sustainability in a warming climate.

## 7 Conclusion

This review underscores phytobiotics as a potent class of nutrigenomic modulators with substantial potential to mitigate HS and its associated physiological, metabolic, and immune disturbances in livestock and poultry. The complex phytochemical composition of these bioactives, particularly polyphenols, flavonoids, and terpenoids, enables coordinated regulation of key molecular networks governing heat shock response, redox balance, and inflammatory signaling. Recent advances in in silico tools, including molecular docking and network pharmacology, have provided valuable mechanistic insight by demonstrating strong interaction of phytocompounds with pivotal targets such as HSP70, NF‐κB, and Nrf2. However, to translate these computational predictions into biologically meaningful and field applicable interventions, future research must adopt integrated multi‐omics approaches. The combined application of transcriptomic, proteomics, metabolomics, and epigenomics will be essential to comprehensively decipher phytobiotic induced regulatory networks, identify molecular biomarkers of thermotolerance, and elucidate species specific metabolic adaptations under HS. Coupling multi‐omics profiling with nutrigenomics will further enable precision optimization of phytobiotic formulations based on host genotype, physiological status, and environmental conditions. In addition, systematic evaluation of dose response relationships, bioavailability, and long‐term safety, under controlled in vitro and in vivo models remains imperative. By integrating computational biology, multi‐omics technologies, and functional validation, phytobiotic based interventions can be transformed into standardized, evidence driven, and sustainable nutritional strategies. Ultimately, this holistic system biology framework will accelerate the development of precision nutrigenomic solutions, thereby enhancing animal resilience, welfare, and productivity under escalating global HS challenges.

## Author Contributions

X.W., A.S. and T.Y. conceived the idea and wrote the initial draft of manuscript; M.A.A. Designed figures and technically revise the manuscript; A.S. Review and edited the final draft. M.A.‐R. Address reviewer comments and perform in silico analysis. All authors read and approve the final draft for submission and publication.

## Funding

This study is supported by the Science and Technology Research Program of Chongqing Municipal Education Commission (Grant No. KJQN202403501, KJQN202303517), National Natural Science Foundation of China No. W2433064. This work was supported by the Deanship of Scientific Research, Vice Presidency for Graduate Studies and Scientific Research, King Faisal University, Al‐Ahsa, Saudi Arabia (Grant NO: KFU260737).

## Ethics Statement

The authors have nothing to report.

## Consent

All authors agreed for submission and publication.

## Conflicts of Interest

The authors declare no conflicts of interest.

## Data Availability

The data that support the findings of this study are available on request from the corresponding author. The data are not publicly available due to privacy or ethical restrictions.
